# Supplementary material for: Compensatory evolution of Pseudomonas aeruginosa’s slow growth phenotype suggests mechanisms of adaptation in cystic fibrosis
Source: Nat Commun. 2021 May 27;12:3186. doi: 10.1038/s41467-021-23451-y (PMC8160344; doi:10.1038/s41467-021-23451-y)
Supplement: Supplementary file 1 — Supplementary Information [file 41467_2021_23451_MOESM1_ESM.pdf]

Compensatory evolution of *Pseudomonas aeruginosa*'s slow growth phenotype suggests mechanisms of adaptation in cystic fibrosis

Ruggero La Rosa, Elio Rossi, Adam M. Feist, Helle Krogh Johansen, Søren Molin

Supplementary Information

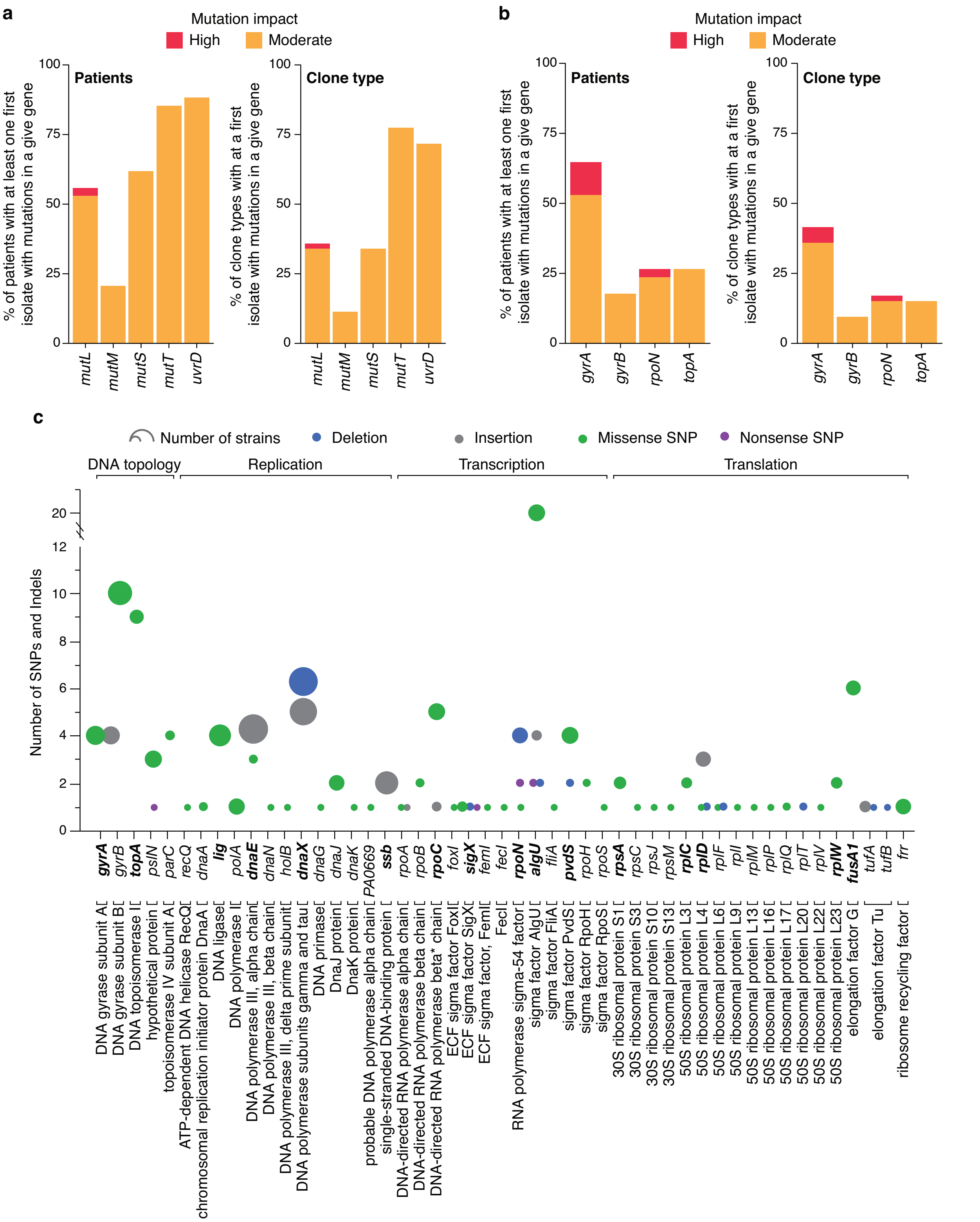

Supplementary Figure 1. Mutations in central component of the cell in a collection of 474 clinical strain of *P. aeruginosa* [1]. (**a**, **b**) Percentage of mutations identified in the first isolate of a give patient and in first isolate of a given clone type for mutations (**a**) in *mutL*, *mutM*, *mutS*, *mutT* and *uvrD* genes and (**b**) *gyrA*, *gyrB*, *topA* and *rpoN* genes. (**c**) Genes encoding

for central components of the cell targeted by mutation during within-patient evolution. In bold are shown the genes in which mutations have been also identified in ALE experiment. Source data are provided as a Source Data file.

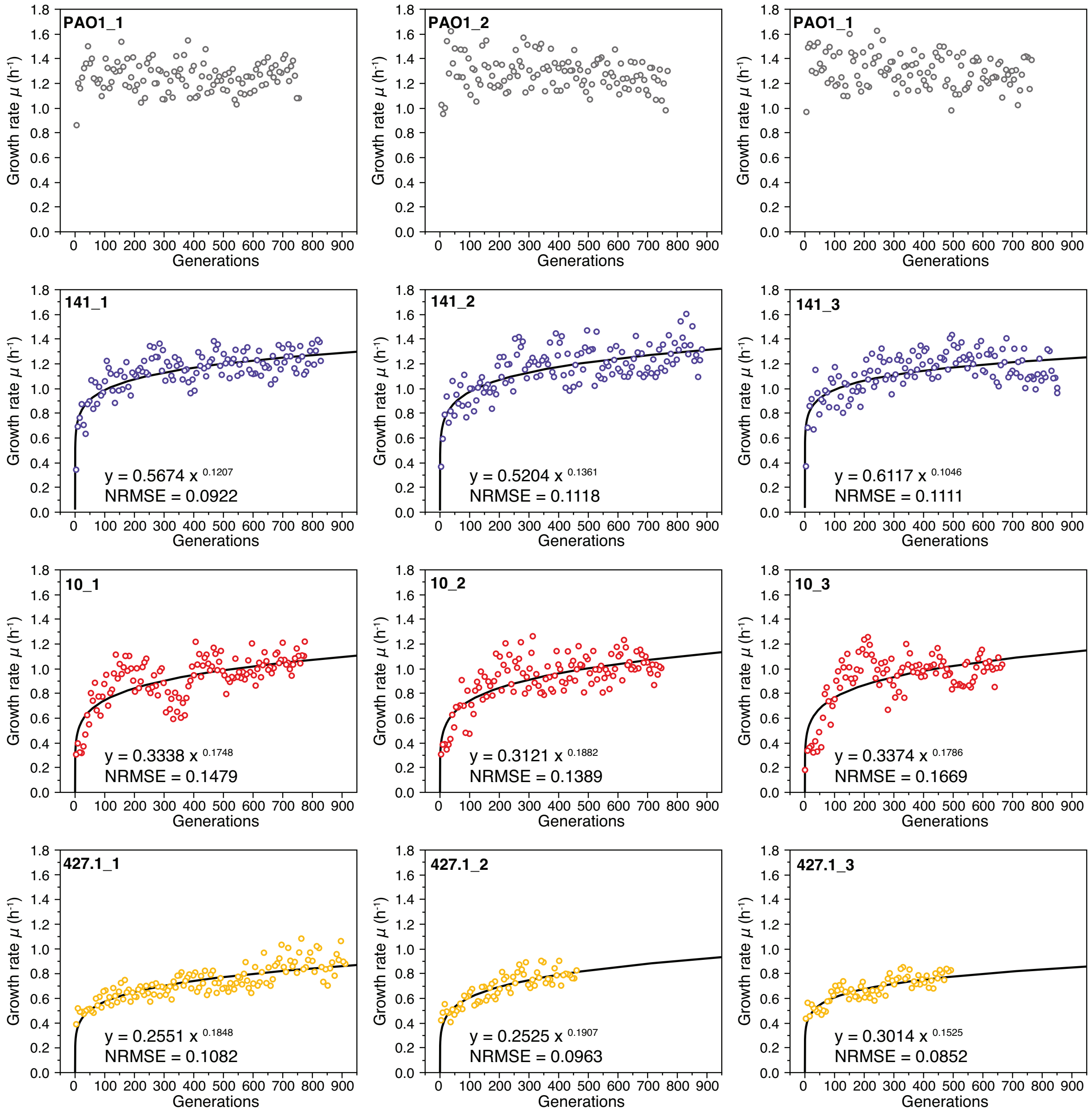

Supplementary Figure 2. Fitness trajectories of each evolving *Pseudomonas aeruginosa* populations. The plots represent the increase in growth rate of each independent populations evolved in parallel. The black curves show the fit of a power-law model. The models, including the normalized root-mean-square error (NRMSE), are indicated in each plot. Source data are provided as a Source Data file.

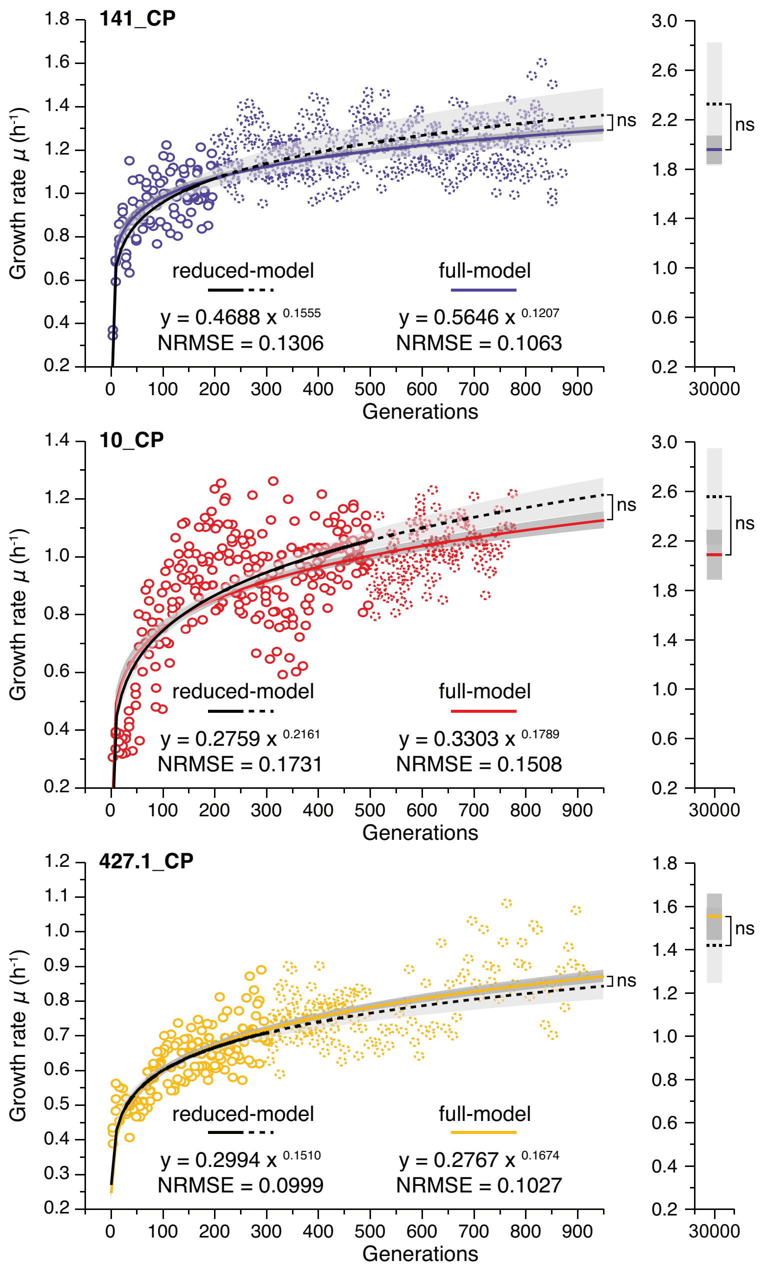

Supplementary Figure 3. Accuracy of the power-law model in simulating posterior distributions of the growth rate of evolving populations. The plot represents the increase in growth rate of each combined population (CP) and the accuracy of the power-law model in predicting the growth rate at 950 and 30,000 generations. The continuous blue (141_CP), red (10_CP) and yellow (427.1_CP) lines and the dark grey areas indicate respectively the fitting of the full power-law model and the 95% confidence interval of the fit computed using the entire dataset of growth rate data as indicated in material and methods and in Figure 1. The black continuous lines, the dashed lines and the light grey areas show respectively the fit of a power-law model, the posterior distribution of the growth rate predicted by the model and the 95% confidence interval of the fit computed using a reduced-model trained with only the growth rate of the first 200, 500 and 300 generations for strains 141, 10 and 427.1,

respectively. The dotted circles indicate the growth rate data not included in the fitting of the reduced model. To evaluate the accuracy of models in simulating posterior distributions of the growth rate, we compared the predictions of the full and reduced model at 950 and 30,000 generations by calculating the statistical significance of the predictions by two-tailed Student's t-test. In all case the *P* value was > 0.05 (ns: not significant) indicating no differences between the posterior distribution of the full and reduced model. The models, including the normalized root-mean-square error (NRMSE), are indicated in each plot.

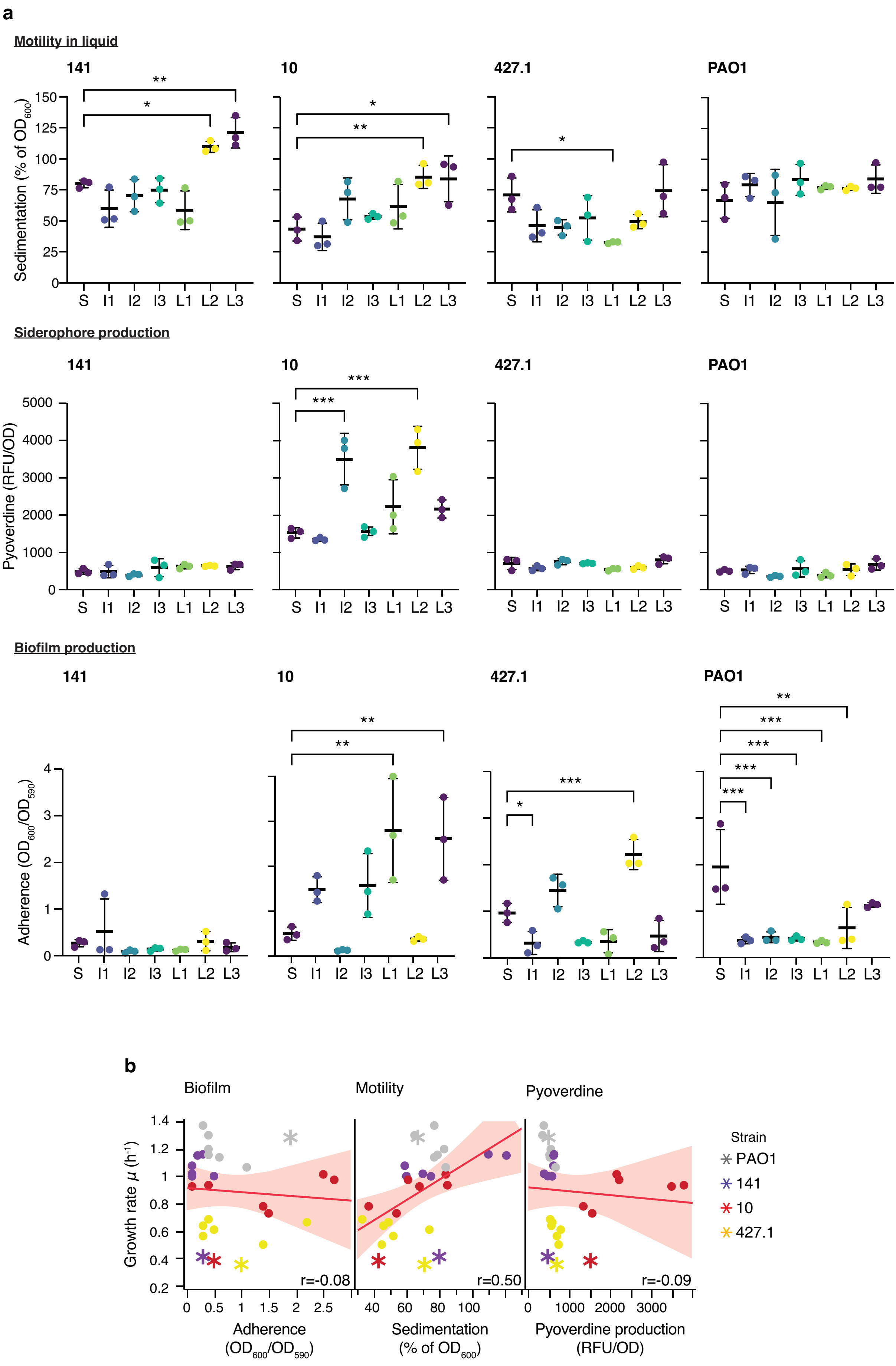

a
Motility in liquid
141
10
427.1
PAO1
Sedimentation (% of OD_{600})
0
25
50
75
100
125
150
S I1 I2 I3 L1 L2 L3
*
**
***
Siderophore production
Pyoverdine (RFU/OD)
0
1000
2000
3000
4000
5000
Biofilm production
Adherence (OD_{600}/OD_{590})
0
1
2
3
4
b
Biofilm
Motility
Pyoverdine
Growth rate μ (h^{-1})
0.2
0.4
0.6
0.8
1
1.2
1.4
r=-0.08
r=0.50
r=-0.09
Adherence (OD_{600}/OD_{590})
0 0.5 1 1.5 2 2.5
Sedimentation (% of OD_{600})
40 60 80 100
Pyoverdine production (RFU/OD)
0 1000 2000 3000
Strain
PAO1
141
10
427.1

Supplementary Figure 4. Phenotypic changes during evolution. (**a**) Motility in liquid was analysed by sedimentation by calculating the ratio of the OD_{600} of a saturated culture after 48h (100%) relative to the same culture incubated for an additional 24h statically [2]. Siderophore production was analysed by measuring the pyoverdine production after 48h of growth in King's B medium [3]. Biofilm production was measured as the ability of the population to adhere to a peg lid of a microtiter plate and to form biofilm. The value represents the ratio between the planktonic cells (OD_{600}) and the biofilm attached to the peg lid (OD_{590}) [1]. In all cases, differences between strains were calculated by one-way ANOVA followed by Tukey's *post hoc* test (*P* values: * < 0.0332; ** < 0.0021; *** < 0.0002; n = 3 biologically independent experiments). Data are presented as mean values +/- SD. (**b**) Pearson's correlation analysis between growth rate and biofilm production, motility and pyoverdine production. The red line and the red area represent the linear relationship between the variables and the 95% confidence interval. The Pearson´s correlation coefficient (r) is indicated at the bottom of each graph. Source data are provided as a Source Data file.

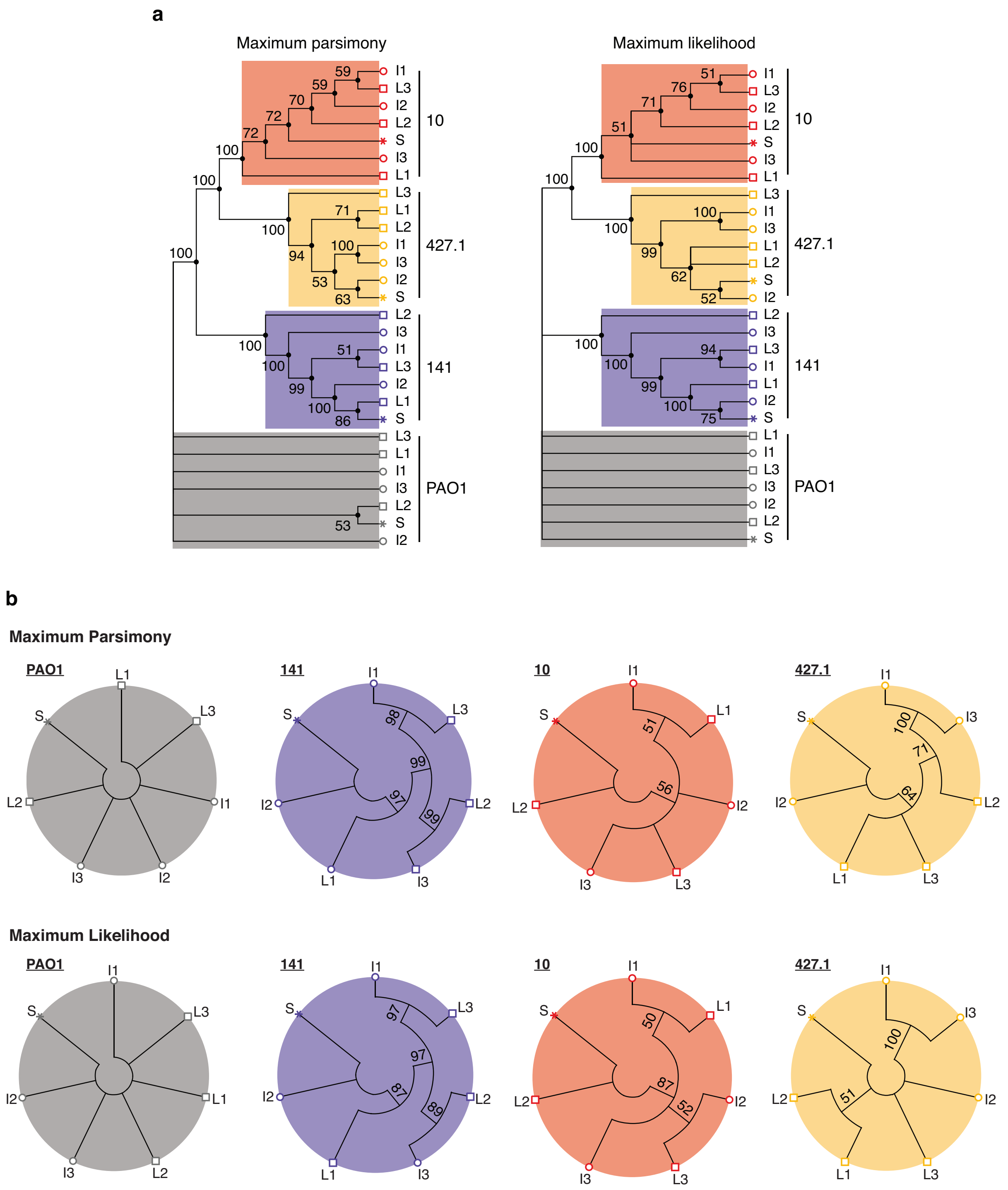

Supplementary Figure 5. Phylogenetic analysis of the evolved strains. (**a**) Maximum parsimony and maximum likelihood reconstruction of the sequenced strains. The tree is based on 8,065 missense and nonsense SNP mutations accumulated during ALE. (**b**) Maximum parsimony and maximum likelihood reconstruction tree for each group of strains PAO1, 141, 10 and 427.1. The trees are based on 134, 5,092, 1,987 and 2,301 missense and nonsense SNP mutations accumulated during ALE respectively in strains PAO1, 141, 10 and 427.1. Branches corresponding to partitions reproduced in less than 50% of

bootstrap replicates are collapsed. The percentages of replicate trees in which the associated taxa clustered together in the bootstrap test (500 replicates) are shown next to the branches.

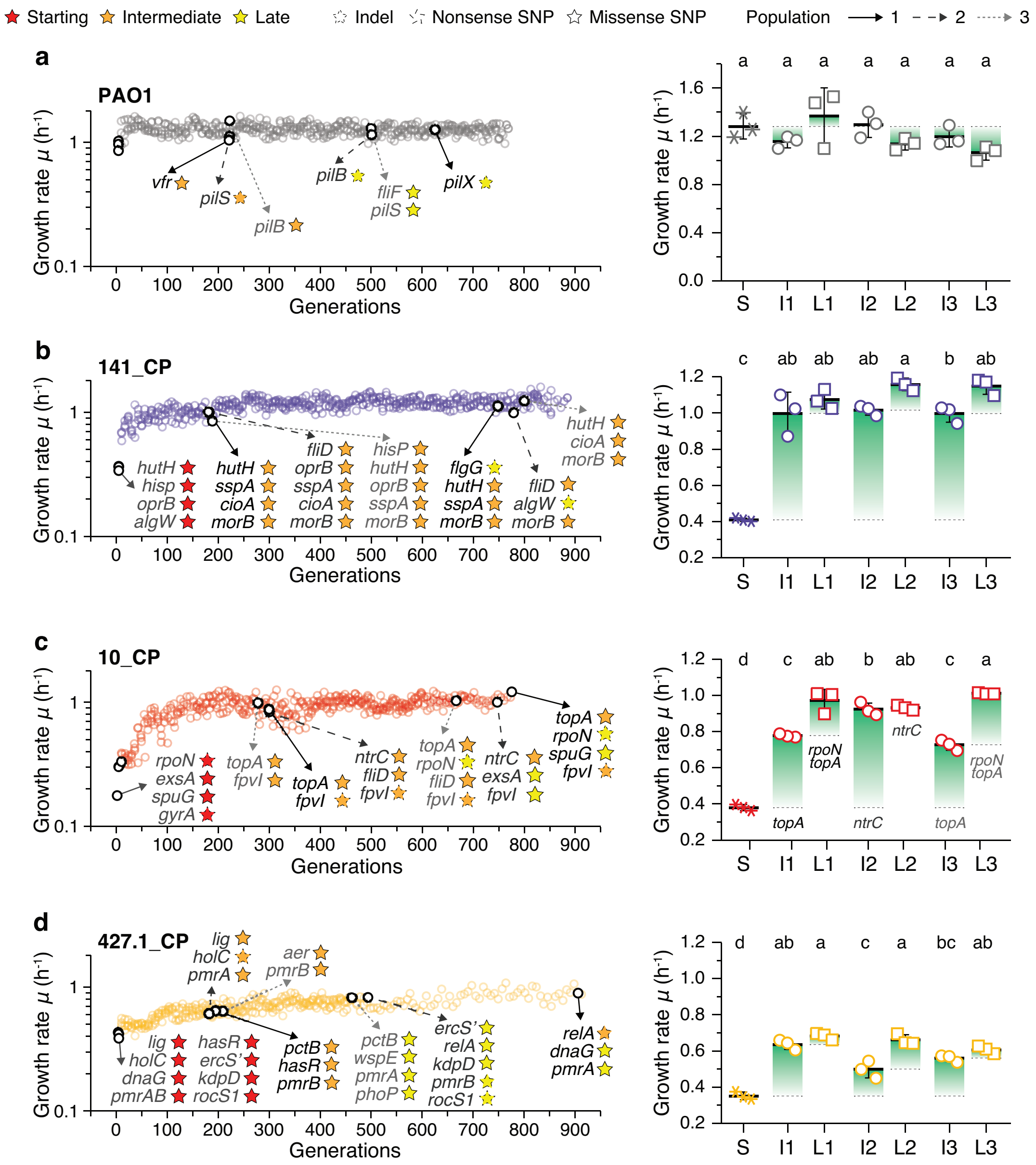

Supplementary Figure 6. Mutations accumulated during ALE. Mutations identified in (**a**) PAO1, (**b**) 141, (**c**) 10 and (**d**) 427.1 strains are shown together with the fitness trajectory of the combined populations (CP). Red, orange and yellow stars, dotted for indels, dashed for nonsense SNP and continuous for missense SNP, indicate mutations present respectively in the starting (S), intermediate (I) and late (L) strains. Mutations in the S strains were identified relative PAO1. Continuous (1), dotted (2) and dashed (3) arrows indicate the population from which I and L strains were isolated. The growth rates for S, I and L strains are reported. Differences between growth rates were calculated by one-way ANOVA followed by Tukey's *post hoc* test. Columns not connected by same letter are significantly different (P value < 0.05; n = 3 biologically independent experiments). Data are presented as mean values +/- SD. Source data are provided as a Source Data file.

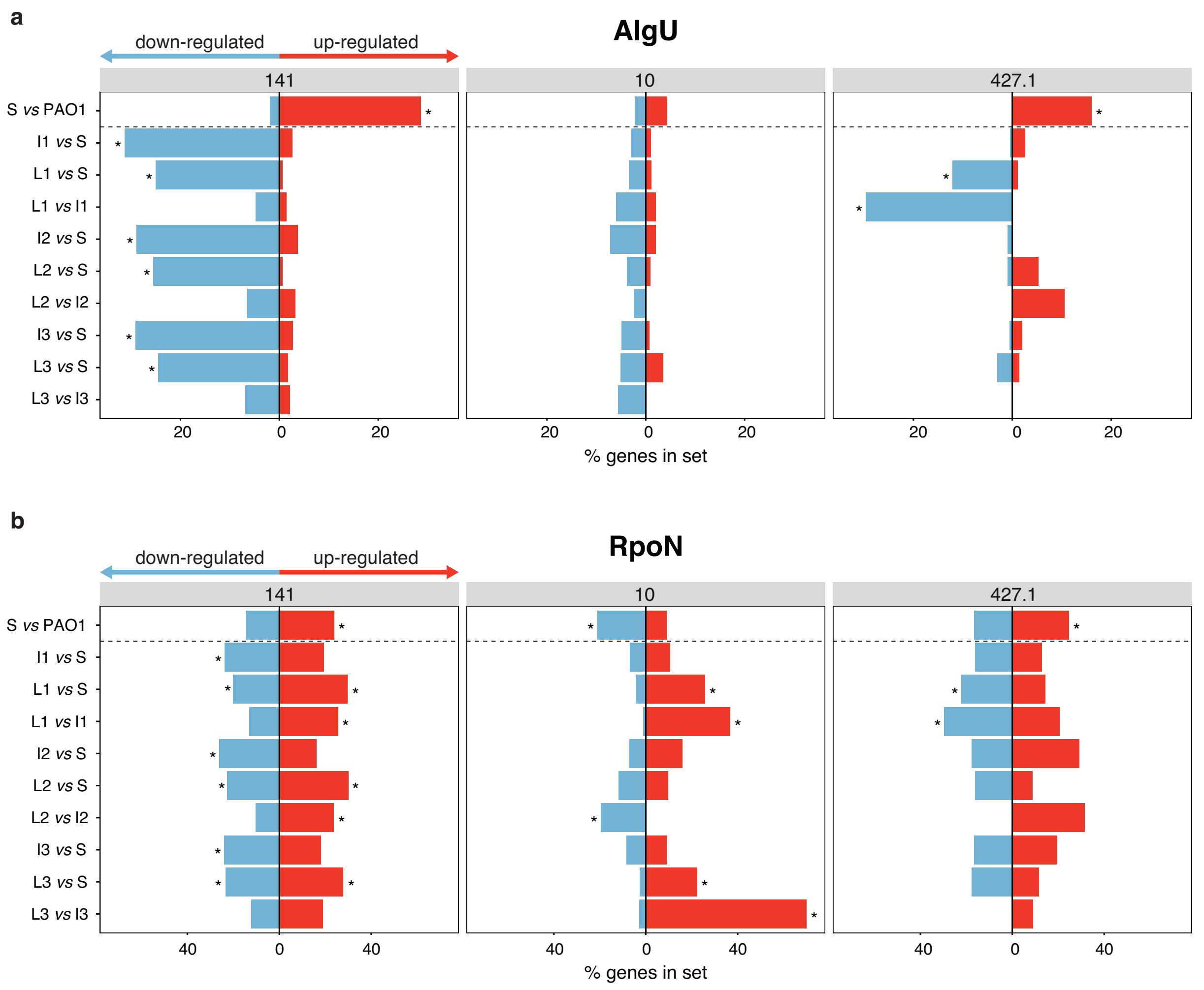

Supplementary Figure 7. Distribution of differentially expressed genes within the (**a**) AlgU and (**b**) RpoN regulons in strains 141, 10 and 427.1. Genes in the regulons were obtained from [4,5]. The percentage of genes upregulated (red bars) or downregulated (blue bars) in each pairwise comparison is reported. Asterisks denote sets of genes significantly enriched (adjusted *P* value ≤ 0.05, hypergeometric test after Bonferroni correction). Source data are provided as a Source Data file.

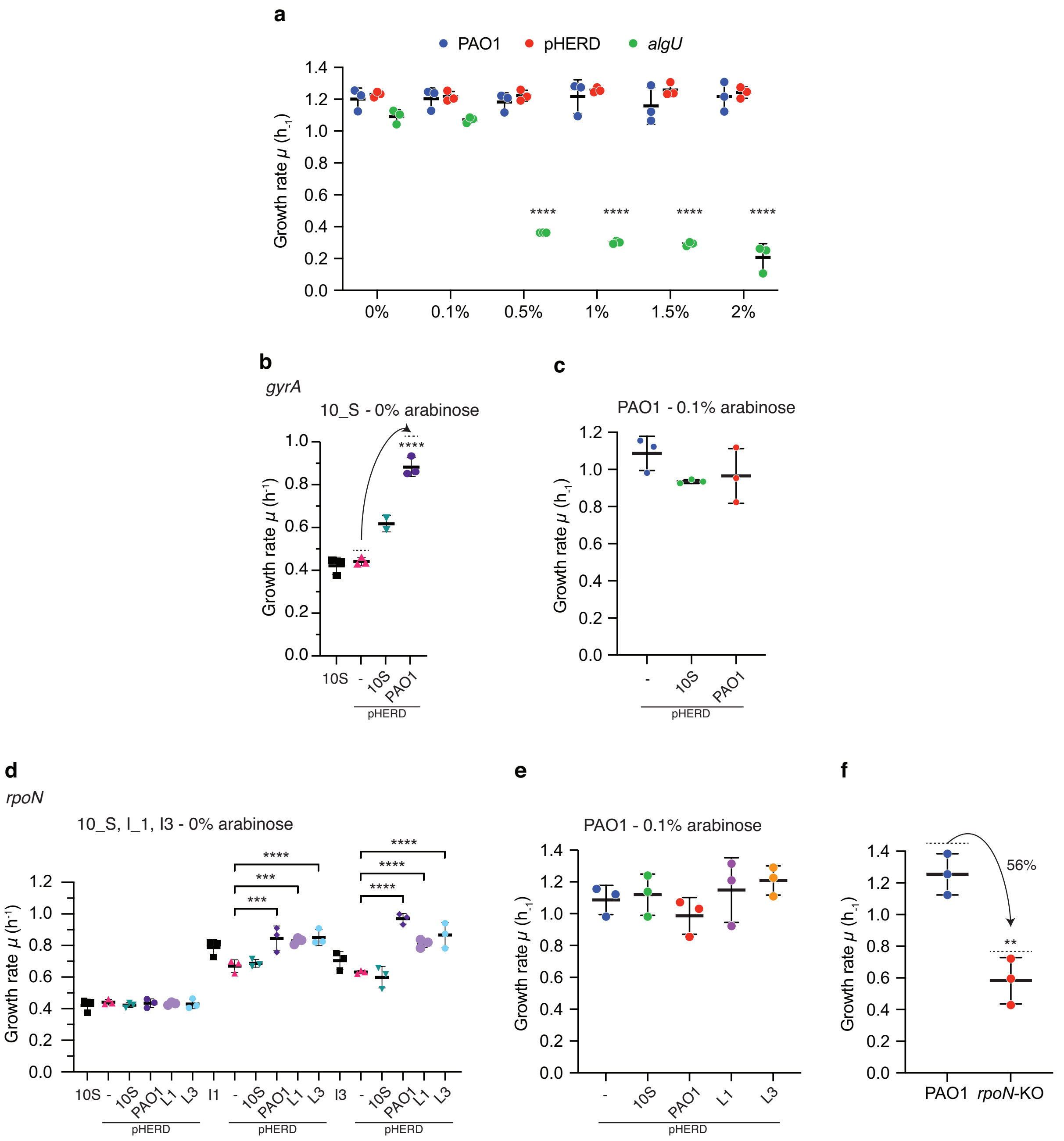

Supplementary Figure 8. Effect of *algU*, *gyrA* and *rpoN* genes expression on PAO1 and 10 strains growth. (**a**) Growth rate reduction in strains PAO1 upon expression of *algU* gene from plasmids p- (empty plasmid) and p*algU* (*algU* gene from strain 141_S) at increasing concentration of arabinose. Statistical significance relative to the absence of inducer (0%) was calculated by Two-Way ANOVA with Dunnett's multiple comparison test (*P* value **** < 0.0001; n = 3 biologically independent experiments). Data are presented as mean values +/- SD. (**b**, **c**) Growth rate changes upon expression of *gyrA* gene from plasmids pHERD: - (empty plasmid), 10_S (*gyrA* gene from strain 10_S) and PAO1 (*gyrA* gene from strain PAO1) in strain (**b**) 10_S in absence of arabinose and in (**c**) PAO1 in presence of 0.1% arabinose. (**d**, **e**) Growth rate changes upon expression of *rpoN* gene from plasmids pHERD:

- (empty plasmid), 10_S (*rpoN* gene from strain 10_S), PAO1 (*rpoN* gene from strain PAO1), L1 (*rpoN* gene from strain 10_L1) and L3 (*rpoN* gene from strain 10_L3) in strains (**d**) 10_S, 10_I1 and 10_I3 in absence of arabinose and in (**e**) PAO1 in presence of 0.1% arabinose. Growth rate differences were calculated relative to the strain expressing the pHERD(-) empty plasmid by One-Way ANOVA with Dunnett's multiple comparison test (*P* values: * < 0.05; ** < 0.0021; *** < 0.0002; **** < 0.0001; n = 3 biologically independent experiments). If not otherwise indicated, differences between strains were not significant. Data are presented as mean values +/- SD. (**f**) Growth rate of a PAO1 derivative mutant strain lacking *rpoN* gene (*rpoN*-KO) [6]. The Difference relative to PAO1 was calculated by Student's t test (*P* value: ** < 0.0021; n = 3 biologically independent experiments). Data are presented as mean values +/- SD. Source data are provided as a Source Data file.

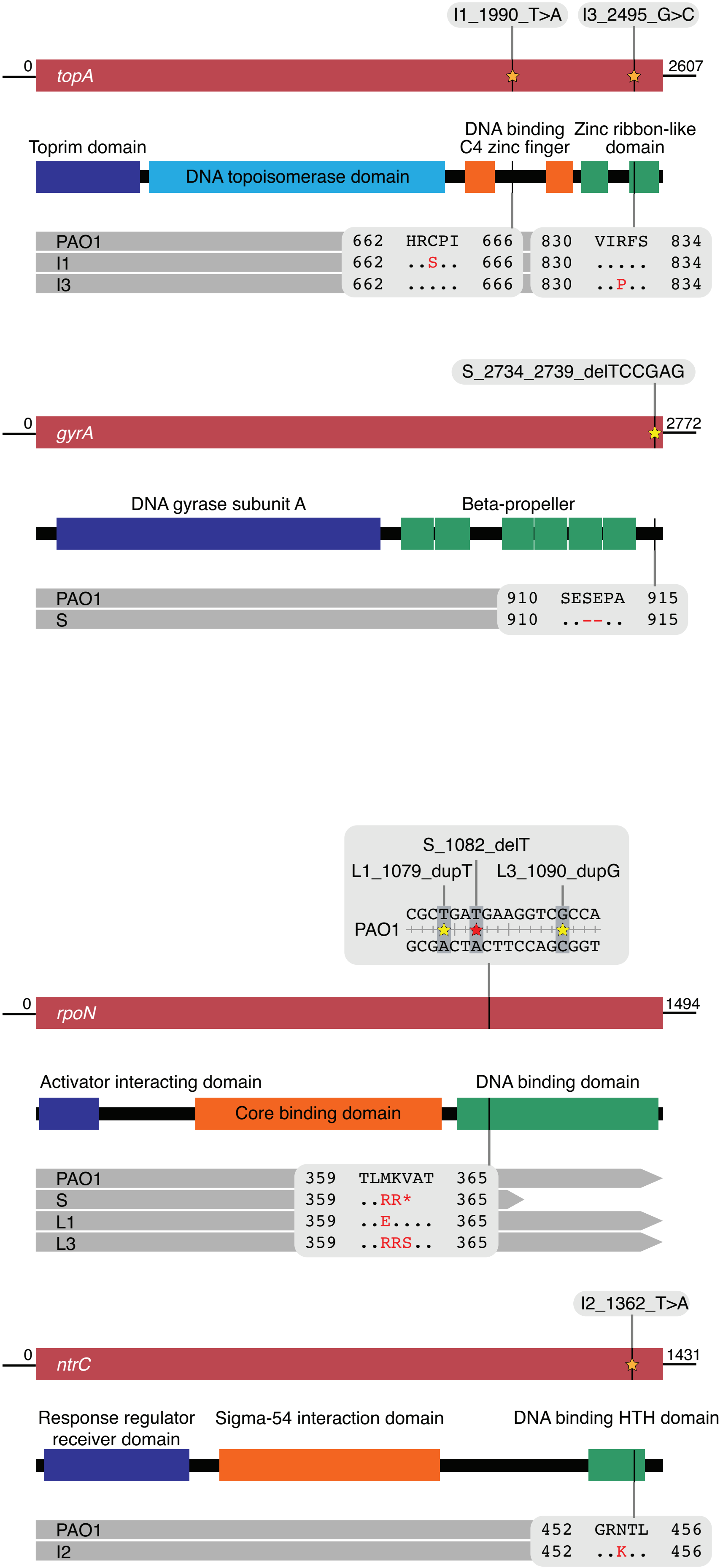

I1_1990_T>A
I3_2495_G>C
0
topA
2607
Toprim domain
DNA topoisomerase domain
DNA binding C4 zinc finger
Zinc ribbon-like domain
PAO1
I1
I3
662 HRCPI 666
662 ..S.. 666
662 666
830 VIRFS 834
830 834
830 ..P.. 834
S_2734_2739_delTCCGAG
0
gyrA
2772
DNA gyrase subunit A
Beta-propeller
PAO1
S
910 SESEPA 915
910 ..--.. 915
S_1082_delT
L1_1079_dupT
L3_1090_dupG
PAO1
CGCTGATGAAGGTCGCCA
GCGACTACTTCCAGCGGT
0
rpoN
1494
Activator interacting domain
Core binding domain
DNA binding domain
PAO1
S
L1
L3
359 TLMKVAT 365
359 ..RR* 365
359 ..E.... 365
359 ..RRS.. 365
I2_1362_T>A
0
ntrC
1431
Response regulator receiver domain
Sigma-54 interaction domain
DNA binding HTH domain
PAO1
I2
452 GRNTL 456
452 ..K.. 456

Supplementary Figure 9. Mapping of the *topA*, *gyrA*, *rpoN* and *ntrC* mutations identified in the ALE evolved strains. Red, orange and yellow stars in the genes (represented in red) indicate mutations (dotted for indels, dashed for nonsense SNP and continuous for missense SNP) present respectively in the starting (S), intermediate (I) and late (L) strains. The functional domains of the proteins are also reported along with the changes in the amino acid sequence.

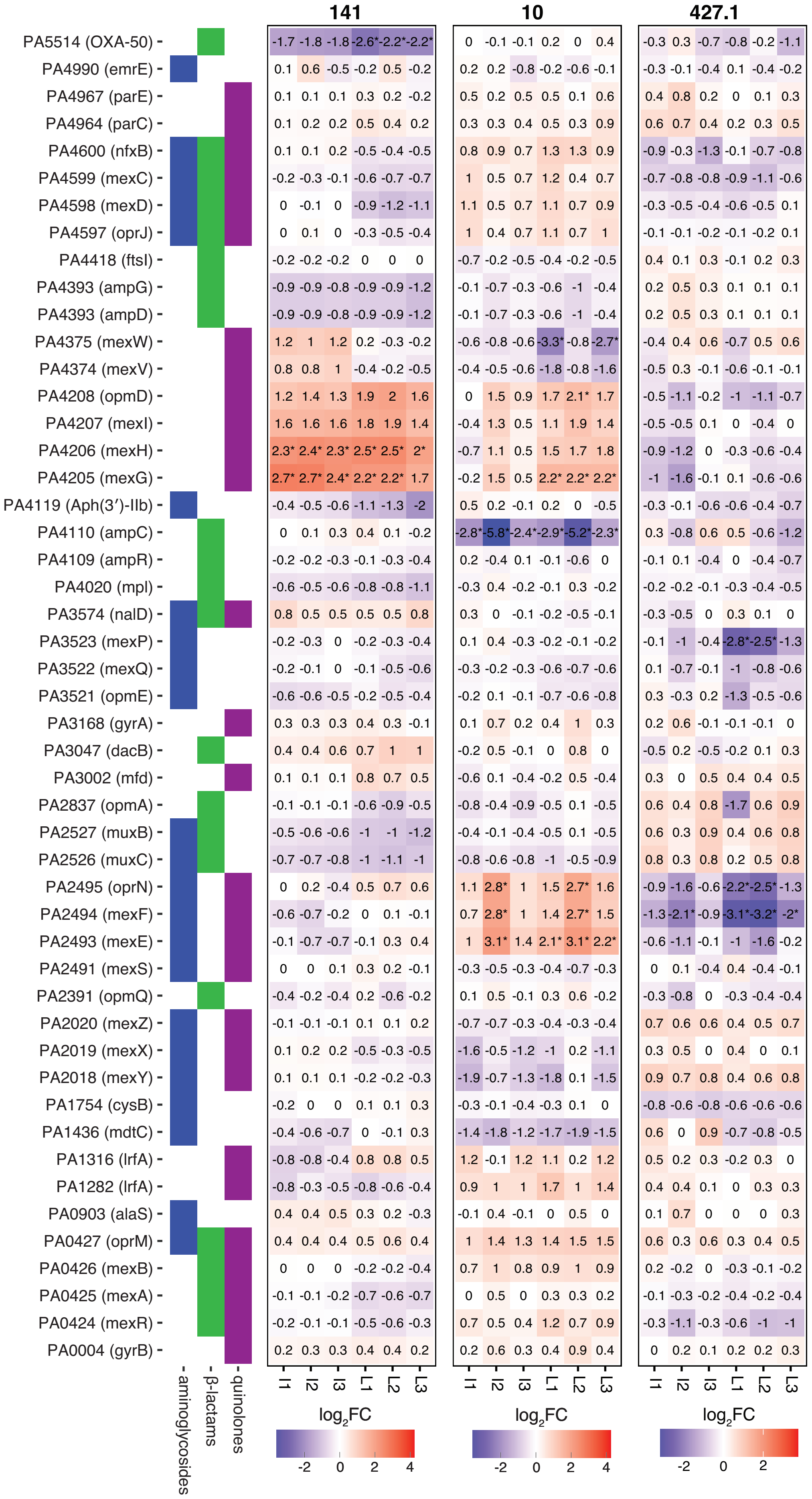
141
10
427.1
PA5514 (OXA-50) -1.7 -1.8 -1.8 -2.6* -2.2* -2.2* | 0 -0.1 -0.1 0.2 0 0.4 | -0.3 0.3 -0.7 -0.8 -0.2 -1.1
PA4990 (emrE) 0.1 0.6 -0.5 -0.2 0.5 -0.2 | 0.2 0.2 -0.8 -0.2 -0.6 -0.1 | -0.3 -0.1 -0.4 0.1 -0.4 -0.2
PA4967 (parE) 0.1 0.1 0.1 0.3 0.2 -0.2 | 0.5 0.2 0.5 0.5 0.1 0.6 | 0.4 0.8 0.2 0 0.1 0.3
PA4964 (parC) 0.1 0.2 0.2 0.5 0.4 0.2 | 0.3 0.3 0.4 0.5 0.3 0.9 | 0.6 0.7 0.4 0.2 0.3 0.5
PA4600 (nfxB) 0.1 0.1 0.2 -0.5 -0.4 -0.5 | 0.8 0.9 0.7 1.3 1.3 0.9 | -0.9 -0.3 -1.3 -0.1 -0.7 -0.8
PA4599 (mexC) -0.2 -0.3 -0.1 -0.6 -0.7 -0.7 | 1 0.5 0.7 1.2 0.4 0.7 | -0.7 -0.8 -0.8 -0.9 -1.1 -0.6
PA4598 (mexD) 0 -0.1 0 -0.9 -1.2 -1.1 | 1.1 0.5 0.7 1.1 0.7 0.9 | -0.3 -0.5 -0.4 -0.6 -0.5 0.1
PA4597 (oprJ) 0 0.1 0 -0.3 -0.5 -0.4 | 1 0.4 0.7 1.1 0.7 1 | -0.1 -0.1 -0.2 -0.1 -0.2 0.1
PA4418 (ftsI) -0.2 -0.2 -0.2 0 0 0 | -0.7 -0.2 -0.5 -0.4 -0.2 -0.5 | 0.4 0.1 0.3 -0.1 0.2 0.3
PA4393 (ampG) -0.9 -0.9 -0.8 -0.9 -0.9 -1.2 | -0.1 -0.7 -0.3 -0.6 -1 -0.4 | 0.2 0.5 0.3 0.1 0.1 0.1
PA4393 (ampD) -0.9 -0.9 -0.8 -0.9 -0.9 -1.2 | -0.1 -0.7 -0.3 -0.6 -1 -0.4 | 0.2 0.5 0.3 0.1 0.1 0.1
PA4375 (mexW) 1.2 1 1.2 0.2 -0.3 -0.2 | -0.6 -0.8 -0.6 -3.3* -0.8 -2.7* | -0.4 0.4 0.6 -0.7 0.5 0.6
PA4374 (mexV) 0.8 0.8 1 -0.4 -0.2 -0.5 | -0.4 -0.5 -0.6 -1.8 -0.8 -1.6 | -0.5 0.3 -0.1 -0.6 -0.1 -0.1
PA4208 (opmD) 1.2 1.4 1.3 1.9 2 1.6 | 0 1.5 0.9 1.7 2.1* 1.7 | -0.5 -1.1 -0.2 -1 -1.1 -0.7
PA4207 (mexI) 1.6 1.6 1.6 1.8 1.9 1.4 | -0.4 1.3 0.5 1.1 1.9 1.4 | -0.5 -0.5 0.1 0 -0.4 0
PA4206 (mexH) 2.3* 2.4* 2.3* 2.5* 2.5* 2* | -0.7 1.1 0.5 1.5 1.7 1.8 | -0.9 -1.2 0 -0.3 -0.6 -0.4
PA4205 (mexG) 2.7* 2.7* 2.4* 2.2* 2.2* 1.7 | -0.2 1.5 0.5 2.2* 2.2* 2.2* | -1 -1.6 -0.1 0.1 -0.6 -0.6
PA4119 (Aph(3′)-IIb) -0.4 -0.5 -0.6 -1.1 -1.3 -2 | 0.5 0.2 -0.1 0.2 0 -0.5 | -0.3 -0.1 -0.6 -0.6 -0.4 -0.7
PA4110 (ampC) 0 0.1 0.3 0.4 0.1 -0.2 | -2.8* -5.8* -2.4* -2.9* -5.2* -2.3* | 0.3 -0.8 0.6 0.5 -0.6 -1.2
PA4109 (ampR) -0.2 -0.2 -0.3 -0.1 -0.3 -0.4 | 0.2 -0.4 0.1 -0.1 -0.6 0 | -0.1 0.1 -0.4 0 -0.4 -0.7
PA4020 (mpl) -0.6 -0.5 -0.6 -0.8 -0.8 -1.1 | -0.3 0.4 -0.2 -0.1 0.3 -0.2 | -0.2 -0.2 -0.1 -0.3 -0.4 -0.5
PA3574 (nalD) 0.8 0.5 0.5 0.5 0.5 0.8 | 0.3 0 -0.1 -0.2 -0.5 -0.1 | -0.3 -0.5 0 0.3 0.1 0
PA3523 (mexP) -0.2 -0.3 0 -0.2 -0.3 -0.4 | 0.1 0.4 -0.3 -0.2 -0.1 -0.2 | -0.1 -1 -0.4 -2.8* -2.5* -1.3
PA3522 (mexQ) -0.2 -0.1 0 -0.1 -0.5 -0.6 | -0.3 -0.2 -0.3 -0.6 -0.7 -0.6 | 0.1 -0.7 -0.1 -1 -0.8 -0.6
PA3521 (opmE) -0.6 -0.6 -0.5 -0.2 -0.5 -0.4 | -0.2 0.1 -0.1 -0.7 -0.6 -0.8 | 0.3 -0.3 0.2 -1.3 -0.5 -0.6
PA3168 (gyrA) 0.3 0.3 0.3 0.4 0.3 -0.1 | 0.1 0.7 0.2 0.4 1 0.3 | 0.2 0.6 -0.1 -0.1 -0.1 0
PA3047 (dacB) 0.4 0.4 0.6 0.7 1 1 | -0.2 0.5 -0.1 0 0.8 0 | -0.5 0.2 -0.5 -0.2 0.1 0.3
PA3002 (mfd) 0.1 0.1 0.1 0.8 0.7 0.5 | -0.6 0.1 -0.4 -0.2 0.5 -0.4 | 0.3 0 0.5 0.4 0.4 0.5
PA2837 (opmA) -0.1 -0.1 -0.1 -0.6 -0.9 -0.5 | -0.8 -0.4 -0.9 -0.5 0.1 -0.5 | 0.6 0.4 0.8 -1.7 0.6 0.9
PA2527 (muxB) -0.5 -0.6 -0.6 -1 -1 -1.2 | -0.4 -0.1 -0.4 -0.5 0.1 -0.5 | 0.6 0.3 0.9 0.4 0.6 0.8
PA2526 (muxC) -0.7 -0.7 -0.8 -1 -1.1 -1 | -0.8 -0.6 -0.8 -1 -0.5 -0.9 | 0.8 0.3 0.8 0.2 0.5 0.8
PA2495 (oprN) 0 0.2 -0.4 0.5 0.7 0.6 | 1.1 2.8* 1 1.5 2.7* 1.6 | -0.9 -1.6 -0.6 -2.2* -2.5* -1.3
PA2494 (mexF) -0.6 -0.7 -0.2 0 0.1 -0.1 | 0.7 2.8* 1 1.4 2.7* 1.5 | -1.3 -2.1* -0.9 -3.1* -3.2* -2*
PA2493 (mexE) -0.1 -0.7 -0.7 -0.1 0.3 0.4 | 1 3.1* 1.4 2.1* 3.1* 2.2* | -0.6 -1.1 -0.1 -1 -1.6 -0.2
PA2491 (mexS) 0 0 0.1 0.3 0.2 -0.1 | -0.3 -0.5 -0.3 -0.4 -0.7 -0.3 | 0 0.1 -0.4 0.4 -0.4 -0.1
PA2391 (opmQ) -0.4 -0.2 -0.4 0.2 -0.6 -0.2 | 0.1 0.5 -0.1 0.3 0.6 -0.2 | -0.3 -0.8 0 -0.3 -0.4 -0.4
PA2020 (mexZ) -0.1 -0.1 -0.1 0.1 0.1 0.2 | -0.7 -0.7 -0.3 -0.4 -0.3 -0.4 | 0.7 0.6 0.6 0.4 0.5 0.7
PA2019 (mexX) 0.1 0.2 0.2 -0.5 -0.3 -0.5 | -1.6 -0.5 -1.2 -1 0.2 -1.1 | 0.3 0.5 0 0.4 0 0.1
PA2018 (mexY) 0.1 0.1 0.1 -0.2 -0.2 -0.3 | -1.9 -0.7 -1.3 -1.8 0.1 -1.5 | 0.9 0.7 0.8 0.4 0.6 0.8
PA1754 (cysB) -0.2 0 0 0.1 0.1 0.3 | -0.3 -0.1 -0.4 -0.3 0.1 0 | -0.8 -0.6 -0.8 -0.6 -0.6 -0.6
PA1436 (mdtC) -0.4 -0.6 -0.7 0 -0.1 0.3 | -1.4 -1.8 -1.2 -1.7 -1.9 -1.5 | 0.6 0 0.9 -0.7 -0.8 -0.5
PA1316 (lrfA) -0.8 -0.8 -0.4 0.8 0.8 0.5 | 1.2 -0.1 1.2 1.1 0.2 1.2 | 0.5 0.2 0.3 -0.2 0.3 0
PA1282 (lrfA) -0.8 -0.3 -0.5 -0.8 -0.6 -0.4 | 0.9 1 1 1.7 1 1.4 | 0.4 0.4 0.1 0 0.3 0.3
PA0903 (alaS) 0.4 0.4 0.5 0.3 0.2 -0.3 | -0.1 0.4 -0.1 0 0.5 0 | 0.1 0.7 0 0 0 0.3
PA0427 (oprM) 0.4 0.4 0.4 0.5 0.6 0.4 | 1 1.4 1.3 1.4 1.5 1.5 | 0.6 0.3 0.6 0.3 0.4 0.5
PA0426 (mexB) 0 0 0 -0.2 -0.2 -0.4 | 0.7 1 0.8 0.9 1 0.9 | 0.2 -0.2 0 -0.3 -0.1 -0.2
PA0425 (mexA) -0.1 -0.1 -0.2 -0.7 -0.6 -0.7 | 0 0.5 0 0.3 0.3 0.2 | -0.1 -0.3 -0.2 -0.4 -0.2 -0.4
PA0424 (mexR) -0.2 -0.1 -0.1 -0.5 -0.6 -0.3 | 0.7 0.5 0.4 1.2 0.7 0.9 | -0.3 -1.1 -0.3 -0.6 -1 -1
PA0004 (gyrB) 0.2 0.3 0.3 0.4 0.4 0.2 | 0.2 0.6 0.3 0.4 0.9 0.4 | 0 0.2 0.1 0.2 0.2 0.3
I1 I2 I3 L1 L2 L3
aminoglycosides
β-lactams
quinolones
log2FC
-2 0 2 4
-2 0 2 4
-2 0 2

Supplementary Figure 10. Expression of genes involved in the resistance to β-lactam, quinolone and aminoglycoside antibiotics. Genes with a Log_2(FoldChange) ≥ I2I and an adjusted p-value ≤ 0.01 in the I and L strains relative to the S strain are considered statistically different and are indicated by an asterisk.

Supplementary References

1. Bartell, J. A. *et al.* Evolutionary highways to persistent bacterial infection. *Nat. Commun.* **10**, 629 (2019).
2. Martínez-García, E., Nikel, P. I., Chavarría, M. & de Lorenzo, V. The metabolic cost of flagellar motion in Pseudomonas putida KT2440. *Environ. Microbiol.* **16**, 291–303 (2014).
3. King, E. O., Ward, M. K. & Raney, D. E. Two simple media for the demonstration of pyocyanin and fluorescin. *J. Lab. Clin. Med.* **44**, 301–7 (1954).
4. Huang, H. *et al.* An integrated genomic regulatory network of virulence-related transcriptional factors in Pseudomonas aeruginosa. *Nat. Commun.* **10**, 1–13 (2019).
5. Schulz, S. *et al.* Elucidation of Sigma Factor-Associated Networks in Pseudomonas aeruginosa Reveals a Modular Architecture with Limited and Function-Specific Crosstalk. *PLoS Pathog.* **11**, 1–21 (2015).
6. Damkiær, S., Yang, L., Molin, S. & Jelsbak, L. Evolutionary remodeling of global regulatory networks during long-term bacterial adaptation to human hosts. *Proc. Natl. Acad. Sci. U. S. A.* **110**, 7766–71 (2013).

Supplementary Table 1. List of primers for *algU* gene overexpression and *gyrA* and *rpoN* genes complementation.

Name	Sequence
algU_usr_(pHERD30T)_F	ATAAATGCUAACCCAGGAACAGGATCAG
algU_usr_(pHERD30T)_R	AGTGCCUCAGGCTTCTCGCAACAAA
pHERD30T_usr_(algU)_F	AGGCACUGGCCGTCGTTTTACAACG
pHERD30T_usr_(algU)_R	AGCATTTAUCAGATCCCATGGGTATGTATATCT
gyrA_usr_(pHERD30T)_F	ATCTGAUAAATGGGCGAACTGGCCAAA
gyrA_usr_(pHERD30T)_R	AGTGCCTUACTCTTCGTTGCCTTCCG
pHERD30T_usr_(gyrA)_F	AAGGCACUGGCCGTCGTTTTACAACG
pHERD30T_usr_(gyrA)_R	ATCAGAUCCCATGGGTATGTATATCTCCTTC
rpoN_usr_(pHERD30T)_F	ATCTGAUAAATGAAACCATCGCTAGTCCTCAA
rpoN_usr_(pHERD30T)_R	AGTGCCUCACACCAGTCGCTTGCGC
pHERD30T_usr_(rpoN)_F	AGGCACUGGCCGTCGTTTTACAACG
pHERD30T_usr_(rpoN)_R	ATCAGAUCCCATGGGTATGTATATCTCCTTC
