# Supplementary material for: Compensatory evolution of Pseudomonas aeruginosa’s slow growth phenotype suggests mechanisms of adaptation in cystic fibrosis
Source: Nat Commun. 2021 May 27;12:3186. doi: 10.1038/s41467-021-23451-y (PMC8160344; doi:10.1038/s41467-021-23451-y)
Supplement: Supplementary file 3 — Description of Additional Supplementary Files [file 41467_2021_23451_MOESM3_ESM.pdf]

**Description of Additional Supplementary Files**

File Name: Supplementary Data 1
Description: Mutations identified during adaptive laboratory evolution in each representative clone.

File Name: Supplementary Data 2
Description: Differentially expressed genes in starting strains of each clone compared to PAO1 starting strains

File Name: Supplementary Data 3
Description: Differentially expressed genes between ALE isolates of PAO1 strain

File Name: Supplementary Data 4
Description: Differentially expressed genes between ALE isolates of clone 141

File Name: Supplementary Data 5
Description: Differentially expressed genes between ALE isolates of clone 10

File Name: Supplementary Data 6
Description: Differentially expressed genes between ALE isolates of clone 427.1
