# Supplementary material for: Compensatory evolution of Pseudomonas aeruginosa’s slow growth phenotype suggests mechanisms of adaptation in cystic fibrosis
Source: Nat Commun. 2021 May 27;12:3186. doi: 10.1038/s41467-021-23451-y (PMC8160344; doi:10.1038/s41467-021-23451-y)
Supplement: Supplementary file 10 — Reporting Summary [file 41467_2021_23451_MOESM10_ESM.pdf]

nature research

Corresponding author(s): Ruggero La Rosa

Last updated by author(s): Apr 19, 2021

Reporting Summary

Nature Research wishes to improve the reproducibility of the work that we publish. This form provides structure for consistency and transparency in reporting. For further information on Nature Research policies, see our Editorial Policies and the Editorial Policy Checklist.

Statistics

For all statistical analyses, confirm that the following items are present in the figure legend, table legend, main text, or Methods section.

n/a	Confirmed	
☐	☒	The exact sample size (n) for each experimental group/condition, given as a discrete number and unit of measurement
☐	☒	A statement on whether measurements were taken from distinct samples or whether the same sample was measured repeatedly
☐	☒	The statistical test(s) used AND whether they are one- or two-sided<br>*Only common tests should be described solely by name; describe more complex techniques in the Methods section.*
☐	☒	A description of all covariates tested
☐	☒	A description of any assumptions or corrections, such as tests of normality and adjustment for multiple comparisons
☐	☒	A full description of the statistical parameters including central tendency (e.g. means) or other basic estimates (e.g. regression coefficient) AND variation (e.g. standard deviation) or associated estimates of uncertainty (e.g. confidence intervals)
☐	☒	For null hypothesis testing, the test statistic (e.g. F, t, r) with confidence intervals, effect sizes, degrees of freedom and P value noted<br>*Give P values as exact values whenever suitable.*
☒	☐	For Bayesian analysis, information on the choice of priors and Markov chain Monte Carlo settings
☐	☒	For hierarchical and complex designs, identification of the appropriate level for tests and full reporting of outcomes
☐	☒	Estimates of effect sizes (e.g. Cohen's d, Pearson's r), indicating how they were calculated

Our web collection on statistics for biologists contains articles on many of the points above.

Software and code

Policy information about availability of computer code

Data collection	Automated adaptive laboratory evolution were performed on a custom Tecan liquid handling robot. Whole genome sequencing and RNA-seq data were collected using Illumina sequencing platform and raw reads generated using the cloud-based Basespace service.

Data analysis	Automated ALE was performed using an in-house script using MATLAB 9.5 (version R2018b). Statistical analyses (ANOVA, Student's t-test, correlation, mixed model and Power-Law model) were carried out in JMP (Version 14.3.0). Bacterial growth rate was calculated using GraphPad Prism (version 8.4.3).<br>Reads derived from whole genome sequencing experiments were processed using Trimmomatic v0.39 and mapped using the BWA aligner v0.7.16a with the MEM algorithm. Picard tools "MarkDuplicates" utility v2.17.0 was used for marking duplicates, and GATK v3.8-0-ge9d806836 to re-align around microindels and to call variants using "HaplotypeCaller" algorithm. Genomes were assembled using the Unicycler assembler v0.4.8. Phylogenetic analyses were carried out in Mega (version 7.0.26).<br>RNA-sequencing reads were processed using Trimmomatic v0.39, SortMeRNA v2.1 and mapped using the BWA aligner v0.7.16a with the MEM algorithm. Reads in genes were counted using htseq-count from the HTSeq v 0.11.2 package.<br>RNA-sequencing data analysis and part of the genomics data analysis are implemented using the R statistical language (R version 4.0.4) using the following freely-available R packages: "ComplexHeatmap v2.4.2", "DESeq2 v1.28.1", "patchwork v1.1.0", "forcats v0.5.0", " "stringr v1.4.0", "dplyr v1.0.2", "purr v0.3.4", "readr v1.4.0", "tidyr v1.1.2", "tibble v3.0.4", "ggplot2 v3.3.2", "tidyverse 1.3.0", "here 1.0.0", "NBClust 3.0". PCA and clustering analysis are performed using the function implemented in base R (prcomp, cor, dist, hclust) or as implemented in the Heatmap function of the ComplexHeatmap package.<br>Gene enrichment analysis is performed using a custom python (python version >= 3.6) script implementing standard term enrichment and statistical testing based on functions available from the modules "scipy v1.5.4", "pandas v1.1.4", "statsmodels v0.12.1".<br>The script is available together with the R code and all raw data necessary to replicate part of the results of the study in the Zenodo repository associated with doi: 10.5281/zenodo.3612820<br>Figures were created in GraphPad Prism (version 8.4.3) and ggplot2 v3.3.2 and further finalized in Adobe Illustrator (version 25.2.1).

For manuscripts utilizing custom algorithms or software that are central to the research but not yet described in published literature, software must be made available to editors and reviewers. We strongly encourage code deposition in a community repository (e.g. GitHub). See the Nature Research guidelines for submitting code & software for further information.

Data

Policy information about availability of data

All manuscripts must include a data availability statement. This statement should provide the following information, where applicable:

- Accession codes, unique identifiers, or web links for publicly available datasets
- A list of figures that have associated raw data
- A description of any restrictions on data availability

Sequencing data that support the findings of this study have been deposited in the EMBL-EBI European Nucleotide Archive (ENA) with the primary accession code PRJEB38310 (https://www.ebi.ac.uk/ena/browser/view/PRJEB38310). Source data are provided with this paper. Virulence factors genes were obtained from the Victors (http://www.phidias.us/victors/), VFDB (http://www.mgc.ac.cn/VFs/) and PseudoCAP (https://www.pseudomonas.com/pseudocap) databases.

Field-specific reporting

Please select the one below that is the best fit for your research. If you are not sure, read the appropriate sections before making your selection.

☐ Life sciences ☐ Behavioural & social sciences ☒ Ecological, evolutionary & environmental sciences

For a reference copy of the document with all sections, see nature.com/documents/nr-reporting-summary-flat.pdf

Ecological, evolutionary & environmental sciences study design

All studies must disclose on these points even when the disclosure is negative.

Study description	Adaptive laboratory evolution of clinical strains of Pseudomonas aeruginosa
Research sample	Three clinical strain and a laboratory strain as control were evolved in three independent adaptive laboratory evolutions (biological replicates). The specific clinical strains were selected since they are representative adapted clinical strains of Pseudomonas aeruginosa belonging to three distinct lineages originally isolated from three independent patients. A detail explanation of the selection is presented in the results section.
Sampling strategy	For each adaptive laboratory evolution a clone from an intermediate and a late time point of evolution were randomly isolated by picking singe colonies from non selective agar plates. A total of six clones in addition to the starting strain were analyzed for each lineage. Evolved strains showed high degree of similarity withing lineage indicating that they are representative of the evolving population.
Data collection	Growth curves were performed on a ELx808 Absorbance Reader (BioTek Instruments, Winooski, VT, USA). E-tests were carried our according to the European Committee on Antimicrobial Susceptibility Testing (EUCAST) guidelines. Biofilm production was assayed on Peg lids (NUNC cat no. 445497) plates and data collected on a ELx808 Absorbance Reader (BioTek Instruments, Winooski, VT, USA). Pyoverdine production data were collected on a Synergy H1 Hybrid Multi-Mode Reader (BioTek Instruments, Winooski, VT, USA).
Timing and spatial scale	For adaptive laboratory evolution, single clones were isolated at an intermediate point after around 450 generations and at a late time point after around 900 generation providing information about the evolutionary trajectories of the evolving populations.
Data exclusions	No data were excluded.
Reproducibility	Adaptive laboratory evolutions were performed in triplicate for each initial strain using independent cultures (biological replicates)

	and genetic data generated from the adapted clones shows great accordance. Potential differences are described in the Result section of the work. Gene expression analysis of selected clones was performed on three independent clones (biological replicates), resulting always in a high level of reproducibility (correlation coefficient > 0.99). All phenotypic experiments were performed on at least three independent clones showing very similar results.
Randomization	Representative clones of the evolved populations were randomly selected by picking single colonies for in vitro experiments.
Blinding	Not relevant. Investigator were not blinded to group allocation.

Did the study involve field work? ☐ Yes ☒ No

Reporting for specific materials, systems and methods

We require information from authors about some types of materials, experimental systems and methods used in many studies. Here, indicate whether each material, system or method listed is relevant to your study. If you are not sure if a list item applies to your research, read the appropriate section before selecting a response.

Materials & experimental systems

n/a	Involved in the study	
☒	☐	Antibodies
☒	☐	Eukaryotic cell lines
☒	☐	Palaeontology and archaeology
☒	☐	Animals and other organisms
☒	☐	Human research participants
☒	☐	Clinical data
☒	☐	Dual use research of concern

Methods

n/a	Involved in the study	
☒	☐	ChIP-seq
☒	☐	Flow cytometry
☒	☐	MRI-based neuroimaging
